# Stable physical activity tracking during children's guided active play

**DOI:** 10.3389/fspor.2022.881664

**Published:** 2022-08-17

**Authors:** Asal Moghaddaszadeh, Urooj Taqvi, Caitlin Lee, Erran Lee, Angelo Belcastro

**Affiliations:** ^1^Pediatric Exercise Physiology Laboratory, School of Kinesiology and Health Science, York University, Toronto, ON, Canada; ^2^Muscle Health Research Centre, Faculty of Health, York University, Toronto, ON, Canada

**Keywords:** play, accelerometer, health, school-aged years, tracking

## Abstract

**Objective:**

Physical activity (PA) decreases during childhood with PA tracking statistics showing moderate coefficients in early childhood moving to poor coefficients in late childhood. The age-related instability of PA tracking is attributed to variations in age-related PA behaviors when quantifying PA in different settings (in/out of school, sports camps, and habitual PA). This study has examined the stability of age-related PA for children (from 7 to 11 years) during self-paced guided active play (GAP) sessions separated by 12 months.

**Methods:**

Children (*n* = 65) recruited from community camps in two consecutive years were assessed for growth and PA participation during GAP sessions (1 h^.^d^−1^ on 2 d^.^wk^−1^) using cooperative games. Accelerometer outputs were used to quantify PA and estimate energy expenditure (EE) and moderate-to-vigorous PA (MVPA). Tracking statistics were assessed by Spearman coefficients (r) and agreement scores for percentile rankings (Kappa (k)) after a 1-year interval.

**Results:**

Grouped data for PA tracking (EE) over the 1-year interval showed strong coefficients (r = 0.88, *p* = 0.001) and moderate agreement scores (k = 0.54). Boys and girls showed similar results. During the 1-year interval, the MVPA tracking coefficient was r = 0.54 (*p* = 0.01) with a moderate k score of 0.47. Age-related PA (EE) tracking coefficients at 7 (r = 0.90), 8 (r = 0.88), 10 (r = 0.82), and 11 (r = 0.83) years showed strong coefficients except at 9 years (r = 0.51).

**Conclusion:**

Children's age-related PA tracking (EE and MVPA) is stable during self-paced GAP assessed over a 1-year interval. Since PA tracking is stable in self-paced GAP, GAP should be included in children's PA intervention strategies to improve health and fitness.

## Introduction

Physical activity (PA) participation and physical fitness (PF) positively impact children and adolescent health- and performance-related outcomes (Poitras et al., 2016; Verswijveren et al., 2018). Despite the importance of PA, surveillance reports for PA behaviors show lower participation rates among school-aged children leading to an increased prevalence of childhood obesity and cardiovascular risk factors (Guthold et al., 2018). As a result, public health strategies emphasize increasing PA behaviors throughout childhood to maintain and improve health outcomes (Bangsbo et al., 2016). To help inform health-related intervention strategies during childhood development, the use of tracking, which refers to the stability or maintenance of a variable's rank over time, has been proposed. Reports show that variables with a high degree of tracking are important for the planning and implementation of intervention strategies to improve health-related outcomes (Malina, 1996, 2001).

Evidence from cross-sectional studies suggests that PA participation decreases with increasing age over the school age and adolescent years (Barnes et al., 2013). Tracking statistics for PA show moderate correlation coefficients in early childhood moving to poor correlation coefficients in the adolescent years (Malina, 1996, 2001; Caldwell et al., 2016). Specifically, moderate tracking correlation coefficients for total PA and moderate-to-vigorous PA (MVPA) were reported for 1-year intervals during the preschool years (<5 years) (Caldwell et al., 2016). The stability of tracking statistics for PA from grades 5 to 8 was reported to be lower for a time at MVPA and energy expenditure (EE) with boys showing stronger tracking than girls (Roth et al., 2018). During late childhood (10–12 years) and adolescence (13–14 years), inter-age correlations for total PA were lowest, ranging from r = 0.03 to r = 0.18, respectively, (Fortier et al., 2001). Explanations used to describe these varied age-related trends for PA tracking statistics include (a) measurement assessment errors when using survey, recall, physiological, and/or movement sensors, (b) study design challenges (i.e., 1 day a week or multiple days per weekday and/or weekend), and (c) the variety in PA responses based on the type or nature of PA opportunities, such as organized vs. unorganized activity and sports vs. play (Jago et al., 2017; Khawaja et al., 2020). In addition to challenges associated with measurement, study design, and type of PA, it has been proposed that tracking studies of PA engagement should not only consider physiological demands of PA but also psychosocial and environmental factors that will contribute to promote PA participation (Malina, 1996, 2001; Pettee-Gabriel et al., 2012; Jago et al., 2017). Free and active play has been reported to provide the physiological, psychological, and environmental contexts that support PA levels from early childhood to adolescents (Truelove et al., 2017). As a result, PA programs that focused on active play might be attractive for use in tracking studies comparing early-middle-late childhood. To date, tracking statistics for PA determined using active play for children and adolescents are not available in the literature.

Guided active play (GAP) is characterized by self-paced, freely chosen, and fun activities incorporating positive role models to encourage children to engage in PA (Truelove et al., 2017). The PA in GAP programs is provided through the use of cooperative (social) games that are classified and clustered into games that exhibit low and high EEs (Howe et al., 2010; Belcastro et al., 2012). In addition, the range of EE and MVPA for cooperative games has been reported to be repeatable from week to week for school-aged children (Belcastro et al., 2012). The physiological requirements for active EE and intensity of PA are provided by the use of cooperative games that increase the total active time and higher levels of MVPA in community-based settings, such as afterschool and community recreation centers (Moghaddaszadeh et al., 2016; Hollis et al., 2017; Khawaja et al., 2020). The psychosocial and environmental correlates of PA are supported by the guided play/fun components of GAP programs that are reported to enhance positive PA behaviors (enjoyment, social interaction, and encouragement) and reduce the negative influences and/or barriers (bullying and low self-efficacy) (Moghaddaszadeh et al., 2016; Truelove et al., 2017; Khawaja et al., 2020). Together these attributes support the use of guided or facilitated active play, using cooperative games as a good candidate for PA tracking during childhood and adolescence. Whether children's participation in the guided active playing of cooperative games shows stable and high tracking statistics that are maintained from childhood to adolescence is not available in the literature.

The purpose of this study was to determine the PA tracking statistics during self-paced GAP sessions separated by 12 months, delivered in a summer day camp setting. PA was assessed for the group and on a gender-specific basis for school-aged children (7–11 years). A cross-sectional analysis of different age groups was conducted to explore the effectiveness of GAP in tracking across childhood since the school ages (7–11 years) cover many of the important age-related stages of development (Malina, 1996, 2001). Tracking statistics for variables were determined with Spearman correlation coefficients and agreement scores (Cohen's *kappa*) for children based on tertial rankings. The identification of stable or non-stable PA tracking statistics from GAP programs, assessed over a 1-year tracking interval, will be relevant for planning children's community-based PA studies/interventions for school-aged children and adolescents.

## Materials and methods

Children participating in this study (*n* = 65 with 28 girls and 37 boys; 7–11 years) were a convenient sample living in a large urban city and registered in a subsidized community summer 5-day camp program. The summer program operated from Monday to Friday (9:00 a.m. to 3:30 p.m.) with a typical day comprised of arts/crafts (25%/day), aquatic activities (10%/day), free time (15%/day), physical activities (25%/day), and social activities (25%/day). The GAP program that consisted of fun, self-paced-based cooperative games were delivered during a 1-h afternoon PA session. With support from the Community Recreation Center, the children's parents/guardians participated in an information/orientation session where they received informed consent material describing procedures, benefits, and the risk of the GAP PA program. Parents/guardians completed a Physical Activity Readiness Questionnaire (PARQ) and provided signed consent for children's participation.

During the initial baseline year and the 1-year follow-up, children were assessed two times for body composition on 2 different days (9:00–10:00 a.m. on Monday and Tuesday) and participated in two GAP sessions on 2 different days (1:30–2:30 p.m. on Tuesday and Thursday). All testing protocols at the baseline and the 1-year follow-up were conducted using the same research team. The procedures used in this study were approved by the University's Human Participants Review Committee.

To complete the GAP sessions, 65 children were randomly divided into two groups of 30–35 and participated in self-paced PA for 1h^.^d^−1^ duration using age-appropriate cooperative games (Belcastro et al., 2015; Truelove et al., 2017; Moghaddaszadeh and Belcastro, 2021). Each group played 5–6 games reported to elicit a range of moderate-to-vigorous intensities (Howe et al., 2010; Belcastro et al., 2012), with one water break introduced halfway through the session. The games included: Giants Wizards Elves, Shipwreck, Octopus, Clothes Pin Tag, Four Corners, Archers Tag, Dr. Dodgeball, Crash, Monkey in the Middle, Handball, Croc-Croc, Line Tag, Toilet Tag, What Time is it Mr. Wolf, Bacon Tag, 4-corner Soccer, 4-corner Basketball, Zombie Tag, Fishes and Whales, Blob Tag, Soccer Baseball, Pizza Oven, The Floor is Lava, and Flip the Fish. The GAP sessions included non-instructional role models (guides) at a ratio of 5:1 for children-to-guide (West and Shores, 2008; Truelove et al., 2017; Moghaddaszadeh and Belcastro, 2021). Experienced undergraduate senior kinesiology majors, with 15 h in a combination of workshops (encouragement strategies; bullying) and simulated children's program delivery (rules of the games and practicing skills), served as positive role models and provided visual encouragement to children to expand their experiences. No instructions and feedback were provided during the sessions, and no child was forced to play games. The cooperative games were conducted in a temperature-controlled (20°C) gymnasium.

Physical activity was quantified during each session using ActiGraph GT3X+ Accelerometers (ACCs). ACCs were placed at the hip with elastic bands and outputs expressed as a vector in counts per 10 s (cnts^.^10 s^−1^). Oxygen consumption (VO_2_) was estimated using a laboratory-generated linear regression equation with a standard error of estimate (SEE) of 0.75 ml^.^kg^−1^min^−1^ for children during treadmill exercise and 3.23 ml^.^kg^−1^min^−1^ when playing active games (Moghaddaszadeh et al., 2018). To classify the volume and intensity of PA, the VO_2_ estimates derived from ACC outputs were used to calculate total EE in a 55-min session (kcal^.^55 min^−1^). Intensity levels, assessed with metabolic equivalents (METs), were predicted by linear regression based on ACC vector outputs, and the percent of time spent at different METs was determined and described as sedentary (0–1.5 MET), very light (1.6–2.9 MET), light (3–3.9 MET), moderate (4–5.9 MET), and vigorous (>6 MET). Finally, the percentage of time spent at each intensity level (expressed as % PA) was determined for each child during all GAP sessions (Belcastro et al., 2015; Moghaddaszadeh et al., 2016; Moghaddaszadeh and Belcastro, 2021). Because of the intermittent (stop-start) nature of PA when children play self-paced cooperative games, classifying time at sedentary activity from movement patterns determined by cutoff points underestimated the metabolic cost of recovery periods occurring between short bursts (Moghaddaszadeh et al., 2018). This increased metabolic response (i.e., metabolic recovery) that occurred at a time when minimal to no movement (i.e., <150 cnts 10 s^−1^) occurred was not captured by the accelerometer. The metabolic cost during recovery has been reported to be important in children's physiological adaptations to exercise (Falk and Dotan, 2006).

Anthropometric variables that included body mass, standing height, and waist circumference were measured two times and averaged as described (Moghaddaszadeh and Belcastro, 2021). Height was measured to the nearest 1.0 cm with a portable stadiometer. Body mass was measured to the nearest 0.2 kg with a standard beam scale. Waist circumference was measured to the nearest 0.5 cm with a standard measuring tape placed above the hip bones. Body mass index (BMI) was calculated as body weight in kilograms divided by height in meters squared (kg^.^ m^2^).

Descriptive and statistical analyses (using SPSSv24) of variables for body composition and levels of PA at baseline and 1-year follow-up were assessed by paired *t*-tests with *p* levels *p* < 0.05, *p* < 0.01, and *p* < 0.001. Tracking was assessed using Spearman rank order correlation coefficients to quantify individual rank stability for each variable between baseline and 1-year follow-up with *p* levels *p* < 0.05, *p* < 0.01, and *p* < 0.001. Correlation coefficients of <0.30 were interpreted as low, those of 0.30–0.60 were interpreted as moderate, and those of >0.6 were interpreted as strong (Caldwell et al., 2016). Children were divided into tertials for each variable (Fortier et al., 2001) and the Cohen's Kappa (*k*) statistic was used to determine the stability within tertials from baseline to 1-year follow-up. The strength of the agreement scores was interpreted using the following schemes: poor **(**_*K*_ = 0.00–0.20), fair (_*K*_ = 0.21–0.40), moderate (_*K*_ = 0.41–0.60), good (_*K*_ = 0.61–0.80), and strong (_*K*_ = 0.81–1.0) (Munoz and Bangdiwala, 1997).

## Results

Children increased body mass, height, BMI, and waist circumference from baseline (BL) to 1-year follow-up (FUI) (Table 1). Girls were younger at baseline (8.4 ± 1.6 years) when compared to boys (9.3 ± 1.7 years; *p* < 0.05). Girls also had statistically less body mass and a lower BMI at baseline (Table 1).

**Table 1:** Participant characteristics described by means and standard deviations (M ± SD), minimum, maximum, and normality with Shapiro-Wilk skewness statistic for selected body composition measures obtained during summer camps at baseline (BL) and 1-year later (FU1).

**Anthropometric measurement**		**Year**	**M ±SD**	**Min**	**Max**	**Skew**
***Total (n** **=** **65)***	Age	BL	8.9 ± 1.7	6.0	11.0	−0.325*
		FU1	9.9 ± 1.7	7.0	12.0	−0.302*
	BM	BL	37.5 ± 12.8	15.5	71.5	0.735*
		FU1	42.6 ± 14.5*	15.8	78.8	0.620*
	HT	BL	140.0 ± 15.6	108	188.2	0.470
		FU1	145.2 ± 15.1*	108	174.0	−0.144
	BMI	BL	18.7 ± 4.0	11.1	30.8	0.895*
		FU1	19.7 ± 4.4*	12.6	33.2	1.27*
	WC	BL	56.4 ± 19.2	46.5	89.5	0.631*
		FU1	67.8 ± 12.4	20.2	97.3	−0.195*
***Girls (n** **=** **28**)*	Age	BL	8.4 ± 1.6^a^	6.0	11.0	−0.007
		FU1	9.3 ± 1.7	7.0	12.0	0.006
	BM	BL	32.7 ± 9.2^a^	18.8	54.3	0.587
		FU1	37.6 ± 11.2*	21.2	61.7	0.455
	HT	BL	135.8 ± 15.9	112.2	188.2	1.39*
		FU1	140.3 ± 14.2*	118.0	168.2	0.315
	BMI	BL	17.5 ± 3.2^a^	11.1	24.2	0.174
		FU1	18.7 ± 2.9*	12.5	24.5	0.241
	WC	BL	63.2 ± 7.7	48.5	81.8	0.293
		FU1	66.4 ± 9.2	52.0	83.8	0.347
***Boys (n** **=** **37)***	Age	BL	9.3 ± 1.7	6.0	11.0	−0.676*
		FU1	10.3 ± 1.7	7.0	12.0	−0.624*
	BM	BL	41.2 ± 14.1	15.5	71.5	0.435
		FU1	46.3 ± 15.7*	15.8	78.8	0.403
	HT	BL	143.1 ± 14.8	108	174.2	−0.163
		FU1	149.0 ± 14.9*	108	177.0	−0.543
	BMI	BL	19.7 ± 4.4	13.3	30.8	0.893*
		FU1	20.4 ± 5.1*	13.6	33.2	1.10*
	WC	BL	67.3 ± 11.6	46.5	89.5	0.461
		FU1	70.2 ± 12.0*	53.0	97.3	0.832*

Body composition measurements included body mass (kg) (BM), height (HT) (cm), body mass index (BMI) (kg^.^ht^2^), and waist circumference (WC) (cm). Comparisons for BL and FU1 and for girls vs boys at BL were *p < 0.05.

Descriptive statistics that included means and standard deviations (SDs), minimum and maximal results, normality statistics, correlation coefficients, and measures of agreement for all PA measures are given in Table 2. Within the grouped data, increases were observed for EE (20%), vigorous PA (VPA) (46%), and MVPA (14%) occurred over the 1-year interval (*p* < 0.05). Gender differences for estimated (EE) during GAP from baseline to 1-year follow-up were increased for girls and boys by 25 and 18%, respectively, with boys having higher EE as compared to girls during the GAP (*p* < 0.05). VPA and MVPA were statistically higher for boys. Tracking coefficients during guide active play sessions were strong for estimated EE (r = 0.88) and moderate for percentages of time at VPA (r = 0.52) and VPA (r = 0.54) for the grouped data. Agreement measures showed moderate Kappa scores for estimated EE and percentage of time at MPA and MVPA from 0.47 to 0.57 (Table 2). Estimated EE tracking coefficients and percentage of time at MVPA were similar for girls and boys during the 1-year interval. Boys had greater tracking coefficients for the percentage of time at VPA (r = 0.55) vs. girls (r = 0.36). Agreement measures showed comparable moderate Kappa scores for estimated EE and percentage of time at MVPA from 0.41 to 0.49 for girls and 0.48 to 0.52 for boys.

**Table 2:** Means and standard deviations (M ± SD), minimum, maximum, Shapiro-Wilk skewness, correlation coefficients, kappa statistics, and classifications for the physical activity measures obtained during a summer day camp program that included guided active play sessions (55 min) at baseline (BL) and 1-year later (FU1).

**Physical activity measurement**		**Year**	**M ±SD**	**Min**	**Max**	**Skew**	**Spearman correlation** **BL vs. FU1**	**Kappa BL vs. FU1 (Class)**
***Total (n** **=** **65)***	EE	BL	207.8 ± 71.3	95.1	373.4	0.536*		
		FU1	250.1 ± 84.6*	93.1	463.4	0.659*	0.88***	0.54 (mod)
	SED	BL	32.7 ± 12.1	9.0	67.0	0.873*		
		FU1	30.1 ± 13.3	6.7	56.5	0.214	0.54**	0.38 (fair)
	MPA	BL	24.6 ± 5.9	9.5	42.1	0.108		
		FU1	25.3 ± 5.8	13.8	40.5	0.056	0.38	0.57 (mod)
	VPA	BL	11.8 ± 5.8	2.6	31.0	0.989**		
		FU1	17.3 ± 7.6*	5.1	40.4	0.889**	0.52**	0.14 (poor)
	MVPA	BL	36.5 ± 8.4	9.7	58.0	−0.110		
		FU1	41.5 ± 11.0*	21.1	69.5	0.402	0.54**	0.47 (mod)
***Girls (n** **=** **28**)*	EE	BL	171.6 ± 50.0^a^	105.7	294.9	0.607		
		FU1	214.8 ± 60.4*	106.2	344.5	0.419	0.82***	0.49 (mod)
	SED	BL	40.2 ± 12.7^a^	18.0	67.0	0.677		
		FU1	36.1 ± 13.5	8.0	56.5	−0.546	0.11	0.22 (fair)
	MPA	BL	23.3 ± 6.7	9.5	38.1	0.033		
		FU1	23.2 ± 5.0	15.7	34.2	0.110	0.10	0.06 (poor)
	VPA	BL	9.8 ± 5.1^a^	2.6	24.1	0.735		
		FU1	13.2 ± 5.5*	5.1	30.8	1.14*	0.36	0.03 (poor)
	MVPA	BL	33.1 ± 8.0^a^	9.7	44.2	−1.06		
		FU1	36.4 ± 9.1	21.1	64.9	0.732	0.49**	0.41 (mod)
***Boys (n** **=** **37)***	EE	BL	234.2 ± 73.0	95.1	373.4	0.162		
		FU1	276.9 ± 91.1*	93.1	463.4	0.371	0.89***	0.53 (mod)
	SED	BL	27.1 ± 8.1	9.0	43.5	0.012		
		FU1	25.7 ± 11.5	6.7	53.2	0.757	0.60***	0.13 (poor)
	MPA	BL	26.6 ± 5.1	16.2	42.0	0.673		
		FU1	26.9 ± 5.9	13.8	40.5	−0.186	0.16	0.11 (poor)
	VPA	BL	13.4 ± 6.1	4.0	31.0	1.11*		
		FU1	20.5 ± 7.6*	9.2	40.4	0.765	0.55***	0.26 (fair)
	MVPA	BL	39.0 ± 7.8	21.6	58.0	0.662*		
		FU1	47.4 ± 10.0*	27.2	69.5	0.389	0.48***	0.48 (mod)

Measurements included estimated energy expenditures (EE) in kilocalories per 55 min. The intensity of the guided active play sessions was described with the percentage of time spent in sedentary (Sed) behaviors [ <1.5 metabolic equivalents (MET), the percentage of time spent in moderate physical activity (MPA; 4–6 MET), the percentage of time spent in vigorous physical activity (VPA; >6 MET), and accumulated percent time in moderate and vigorous physical activity (MVPA; >4 MET)].

^*^p < 0.05:

^**^p < 0.01;

^***^p < 0.001.

Girls vs. boys at BL were ^a^ = p < 0.05.

Means and SDs, correlation coefficients, kappa scores, and classifications for EEs and PA intensity analyzed over 1-year intervals starting at 7, 8, 9, 10, and 11 years (*n* = 65) are shown in Table 3. The age-related changes in EE showed that the older children (10 and 11 years) had statistically greater EE than the younger children (7–9 years; Table 3). None of the intensity-related components was different across the age ranges. When considering tracking statistics, strong correlation coefficients for EE were observed at each age with younger children showing higher coefficients except for the 9-year-old children, the Kappa statistics for EE showed moderate agreement scores for the older children, while the younger children had fair agreements scores except for the 8-year-old children. The tracking of sedentary behavior during the GAP program demonstrated the lowest correlation coefficients at 7 and 8 years (average of r = 0.25) and the strongest correlation coefficients at 10–11year (average of r = 0.70). Kappa scores were classified as fair across the age range with the exception of children at 9 years that demonstrated a poor agreement score. Regarding the percentage of time at VPA of PA, children demonstrated stronger tracking coefficients at 7 years (r = 0.78), which was lowered to r = 0.42 at 11 years. Kappa scores for children at 7 years were classified as good, with poor classifications noted for children at 8–10 years. Regarding the time at MVPA, statistically significant coefficients were observed for children at 7–10 years, but not for those at 11 years (Table 3).

**Table 3:** Age-related means and standard deviations (M ± SD), correlation coefficients, kappa statistics, and kappa classifications for the physical activity measures obtained during summer camps guided active play sessions (55 min) at baseline (BL) and 1-year later (FU1).

***Physical activity measurement***	**EE**		**SED**		**MPA**		**VPA**		**MVPA**	
**Year**	**BL**	**FU1**	**BL**	**FU1**	**BL**	**FU1**	**BL**	**FU1**	**BL**	**FU1**
	***7 years (G4:B5)***									
M ± SD	171.5 ± 42.0	200.0 ± 49.3*	29.2 ± 9.7	29.1 ± 12.4	25.3 ± 8.6	26.4 ± 4.7	13.6 ± 3.5	18.2 ± 6.6*	38.9 ± 6.0	44.6 ± 7.4*
Spearman's Correlation	0.90***		0.27		0.35		0.78**		0.55*	
Kappa (Class)	0.33 (fair)		0.33 (fair)		0.17 (poor)		0.67 (good)		0.17 (poor)	
	***8 years (G10:B6)***									
M ± SD	175.2 ± 43.9	221.4 ± 70.4	35.8 ± 15.7	37.1 ± 12.9	24.5 ± 10.2	22.7 ± 5.2	13.1 ±10.1	15.3 ± 8.0	37.6 ± 14.5	38.0 ± 12.0
Spearman's Correlation	0.91***		0.12		0.25		0.51*		0.72**	
Kappa (Class)	0.43 (mod)		0.21 (fair)		0.07 (poor)		0.19 (poor)		0.30 (fair)	
	***9 years (G6:B7)***									
M ± SD	195.6 ± 58.5	226.1 ± 44.2	37.6 ± 14.9	28.2 ± 13.0	22.2 ± 4.6	26.8 ± 5.9*	11.3 ± 5.3	15.5 ± 7.2*	33.5 ± 8.3	42.4 ±12.1**
Spearman's Correlation	0.51		0.56*		0.74**		0.48		0.82***	
Kappa (Class)	0.31 (fair)		0.17 (poor)		0.07 (poor)		0.07 (poor)		0.18 (poor)	
	***10 years (G3:B8)***									
M ± SD	224.8 ± 44.0	278.4 ± 70.1^a^	32.9 ± 10.1	32.8 ± 15.1	24.3 ± 3.6	24.1 ± 4.4	10.7 ± 4.1	17.3 ± 5.5**	36.5 ± 6.6	46.5 ± 14.3***
Spearman's Correlation	0.82***		0.60*		0.29		0.41		0.76*	
Kappa (Class)	0.45 (mod)		0.31 (fair)		0.09 (poor)		0.10 (poor)		0.21 (fair)	
	***11 years (G5:B11)***									
M ± SD	273.9 ± 71.0	323.3± 73.4*^b^	30.5 ± 10.2	26.0 ± 12.8*	25.3 ± 3.8	25.9 ± 7.0	11.2 ± 4.9	20.6 ± 10.2***	36.5 ± 6.6	46.5 ± 14.3***
Spearman's Correlation	0.83***		0.79**		0.05		0.42		0.50	
Kappa (Class)	0.41 (mod)		0.21 (fair)		0.10 (poor)		0.21 (fair)		0.42 (mod)	

Measurements included estimated energy expenditures (EE) in kilocalories per 55 min. The intensity of the guided active play sessions included the percentage of time spent in sedentary (SED) behaviors [ <1.5 metabolic equivalents (MET), the percentage of time spent in moderate physical activity (MPA; 4–6 MET), the percentage of time spent in vigorous physical activity (VPA; >6 MET), and accumulated time in moderate and vigorous physical activity (MVPA; >4 MET)].

^*^p < 0.05:

^**^p < 0.01;

^***^p < 0.001.

Age comparisons ^a^ = p < 0.05 for 10 vs. 7–11 ^b^ = p < 0.05 for 11 vs. 7–10 ^b^ = p < 0.05.

## Discussion

The aim of the study was to determine PA levels during GAP and investigate PA tracking statistics over a 1-year interval from early to late childhood. PA levels for GAP were assessed during 55-min of self-paced playing of cooperative games two times at baseline and two times at 1-year follow-up. Previous findings have reported that PA and intensity of PA show low-to-moderate tracking coefficients during childhood with inter-age correlations higher in early childhood and dropping in late childhood with boys tracking higher than girls (Malina, 1996, 2001; Caldwell et al., 2016). The lower PA tracking statistics throughout childhood are more evident for longer (6, 9, and/or 12 years) as compared to shorter (1, 2, or 3 year) age intervals (Malina, 2001). To our knowledge, no reports have investigated tracking of PA during GAP using total estimated EE and percent time at different PA intensities (MVP, VPA, and MVPA) in children in early, middle, and late childhood. Our results show strong levels of tracking for EE, VPA, and MVPA over a 1-year interval for school-aged boys and girls participating in self-paced games in a GAP format. Therefore, our play-based results open up the need to consider the benefits of tracking children's PA in settings that will encourage and engage children in positive PA behaviors rather than restrict observations to habitual activity (Moghaddaszadeh et al., 2016; Truelove et al., 2017; Khawaja et al., 2020).

Changes in PA levels quantified by self-reporting tend to show declines in early childhood (5.7–7.9 years) (Crane et al., 2018), middle-late childhood, and adolescents (8.8–12.1 years) (Fortier et al., 2001; McMurray et al., 2003). Other studies had similar results when using previous day recall (Pate et al., 1999) and 3-day sweat rates (10.5–11.5 years) (Janz et al., 2000). In the present study, accelerometer-quantified PA levels during GAP showed increases in estimated EE (kcal^.^55 min^−1^), %VPA and %MVPA for children (6–11 years) during a 1-year interval. Gender differences tended to show similar increases for boys and girls in estimated EE and %VPA, but a lower %MVPA for girls. In addition to the grouped data, aged-related PA levels showed an increase in estimated EE for children at 7–11 years over the 12 months. The age-related changes and gender differences noted in this study suggest that older children engage in more PA than younger children and boys engage in more PA than girls during active play. Our findings are consistent with previous work during GAP, which suggests increases in total PA of 22% for all children over a 2-year interval, with boys increasing by 27% and girls by 17% when participating in a children's lifestyle and energetics program (Kelly et al., 2007).

There are limited reports available for PA tracking of children from early-middle-late childhood with GAP. One study reported a lower PA tracking coefficient (r = 0.37) over a 2-year interval during early childhood participation in a lifestyle and energetic program (Kelly et al., 2007). The majority of studies have assessed tracking statistics over several days/hours during habitual PA and/or organized sports participation with a few studies using a play-based approach. Furthermore, the variations in tracking statistics reported in previous PA studies have used a variety of age groups, tracking intervals, methods, and measurement tools (Jago et al., 2017; Khawaja et al., 2020). It has been suggested that attention must be given to PA settings/approaches when comparing tracking statistics to assess children's PA behaviors (Malina, 1996, 2001). Our results for PA tracking of estimated EE during GAP revealed strong coefficients (r ≥ 0.8) and moderate agreement (≈0.50) scores for grouped school-aged children. For PA intensities, the %VPA and %MVPA showed moderate tracking coefficients of r = 0.52 and r = 0.54, respectively. Gender differences showed that boys had higher levels of EE, %VPA, and %MVPA with less sedentary time during the GAP as compared to girls. Boys and girls showed strong tracking and moderate agreement scores for EE and %MVPA, with only the boys showing statistically greater %VPA tracking. Age-related EE increased from 7 to 11 years with stable tracking coefficients observed at each age, except for 9 years. The tracking coefficients for %MVPA and sedentary time showed increased stability with increasing age, while coefficients for %VPA showed less stability from 7 to 11 years. These findings that are analyzed over a 1-year tracking interval and a cross-section of children in early, middle, and late childhood provide “proof-of-concept” for tracking PA using self-paced playing of cooperative games in a GAP format to promote PA participation. These findings demonstrate the importance and practicability of children's self-paced GAP in providing consistent stable PA tracking throughout middle and late childhood. Finally, evidence suggests that children are more likely to participate in PA with a freely chosen guided play environment, which aligns with the self-paced, cooperative, and non-competitive nature of this program (Pettee-Gabriel et al., 2012; Jago et al., 2017). The pattern of the traditional exercise programs tends to be prolonged and continuous, whereas children's normal activity pattern is in short bursts, approximately 3 s in duration. The facilitated play has been reported to promote higher levels of PA as compared to the traditional exercise programs (Moghaddaszadeh et al., 2016; Truelove et al., 2017). The reported increase in total PA with cooperative, non-competitive games is also likely attributed to the program's encouragement of play and aspects of child wellbeing. The program allows children to experience the joys of movement through the playing of various games and the language used to promote PA participation during play encourages movement (Pyle and Danniels, 2017; Truelove et al., 2017; Moghaddaszadeh and Belcastro, 2021).

There are some limitations to the interpretation of these results. First, although tracking studies can occur over longer time intervals, a 1-year interval reflects an important proportion of a child's life. Many important growth and developmental processes occur, which may elicit changes in PA behaviors during the 12 months. Investigating longer time intervals would increase the potential impact of these results. Second, a longitudinal study over many years and with many more children would enhance the generalizability of the results. Although the study only included 65 children over a 1-year interval, it tracked children across the school ages (7–11 years) that cover many of the important age-related stages of development. Although our sample size was not sufficiently large to stratify the results by age, preliminary analyses showed age-appropriate differences for growth variables across the groups. Hence, we believe that our findings are based on a representative sample of children covering early, middle, and late childhood. Third, our analyses did not contain a control group. In defense, study designs used in “real-world” settings, such as community center summer day camps, are challenging and often associated without the benefits of a control group with the naturally emerging situations requiring use of quasi-experimental study designs. Although this approach may comprise the generalizability of our results, we view these results as exploratory and serve as a “proof-of-concept” for children's play-based studies. Fourth, PA outputs for GAP may be affected by the summer camp environment and the nature of the games played. Our study used the same research team for each session over the 1-year interval. The success of the GAP sessions was influenced by a respectful partnership developed between the research team and the community center administration. All GAP sessions were delivered with a common series of games, reported to be reliable and repeatable over time (Belcastro et al., 2012, 2015). Finally, we used estimated MET levels derived from accelerometer outputs and not vector cut-points to classify children's PA intensity during GAP (Moghaddaszadeh and Belcastro, 2021). Currently, there are no sets of PA cutoff thresholds for a short burst, frequent start-and-stop PA behaviors that occur with children playing cooperative games. This is not a trivial matter, and we reported the increased metabolic demands that persist when children show very low ACC vector outputs (Moghaddaszadeh et al., 2018). Notwithstanding these limitations, the need to confirm the stability of PA tracking for children's play should consider the use of a larger sample size in follow-up years to accurately assess the influences of developmental and maturity stages and include several follow-up years with increased intervals between ages.

In summary, the evidence suggests that children are more likely to participate in PA within an unstructured play environment consisting of a self-paced, cooperative (social), and non-competitive nature of GAP. This approach to track children's PA participation may promote stronger relationships between cardiometabolic risk factors during development. With the growing obesity epidemic, it is important to rephrase the way in which PA is promoted, in a way that children and parents find meaningful.

## Data availability statement

The raw data supporting the conclusions of this article will be made available by the authors, without undue reservation.

## Ethics statement

The studies involving human participants were reviewed and approved by Office of Research Ethics (ORE), Human Participants Review Committee, York University. Written informed consent to participate in this study was provided by the participants' legal guardian/next of kin.

## Author contributions

AB and AM for conception and design of research. AM, UT, EL, and CL delivered the physical activity program and collected the data. UT, EL, and CL analyzed the data and prepared tables/figures. AB and AM interpreted results of experiments and prepared the initial manuscript. All authors approved the final version of the manuscript.

## Funding

Funding for this project was provided by the Faculty of Health Small Grants Program at York University.

## Conflict of interest

The authors declare that the research was conducted in the absence of any commercial or financial relationships that could be construed as a potential conflict of interest.

## Publisher's note

All claims expressed in this article are solely those of the authors and do not necessarily represent those of their affiliated organizations, or those of the publisher, the editors and the reviewers. Any product that may be evaluated in this article, or claim that may be made by its manufacturer, is not guaranteed or endorsed by the publisher.

## References

[B1] Bangsbo J. Krustrup P. Duda J. Hillman C. Andersen L. B. Weiss M.. (2016). The Copenhagen Consensus Conference 2016: children, youth, and physical activity in schools and during leisure time. Br. J. Sports Med. 50, 1177–1178. 10.1136/bjsports-2016-096325 27354718 PMC5036221

[B2] Barnes J. D. Colley R. C. Borghese M. Janson K. Fink A. Tremblay M. S. (2013). Results from the active healthy kids Canada 2012 report card on physical activity for children and youth. Pediatr. Child Health. 18, 301–304. 24421697 PMC3680251

[B3] Belcastro A. Mofidi D. M. Ahmadi Y. Moghaddaszadeh A. (2015). Correlates of psychosocial health-related quality of life measures for normal weight and obese children participating in an active play program. J. Child Adolesc. Behav. 3, 242. 10.4172/2375-4494.1000242

[B4] Belcastro A. N. Morrison K. S. Hicks E. Matta H. (2012). Cardiorespiratory and metabolic responses associated with children's physical activity during self-paced games. Can. J. Physiol. Pharmacol. 90, 1269–1276. 10.1139/y2012-106 22913550

[B5] Caldwell H. A. T. Proudfoot N. A. King-Dowling S. Cristofaro N. A. Cairney J. Timmons B. W. (2016). Tracking of physical activity and fitness during the early years. Appl. Physiol. Nutr. Metab. 41, 504–510. 10.1139/apnm-2015-0338 27045869

[B6] Crane J. R. Naylor P.-J. Temple V. A. (2018). The physical activity and sedentary behaviour patterns of children in kindergarten and grade 2. Children. 5, 131. 10.3390/children5100131 30241367 PMC6210440

[B7] Falk B. Dotan R. (2006). Child-Adult differences in the recovery from high-intensity exercise. Exerc. Sport Sci. Rev. 34, 107–112. 10.1249/00003677-200607000-00004 16829737

[B8] Fortier M. D. Katzmarzyk P. T. Malina R. M. Bouchard C. (2001). Seven-year stability of physical activity and musculoskeletal fitness in the Canadian population. Med. Sci. Sports Exerc. 33, 1905–1911. 10.1097/00005768-200111000-00016 11689742

[B9] Guthold R. Stevens G. A. Riley L. M. Bull F. C. (2018). Worldwide trends in insufficient physical activity from 2001 to 2016: a pooled analysis of 358 population-based surveys with 1·9 million participants. Lancet Global Health. 6, e1077–e1086. 10.1016/S2214-109X(18)30357-7 30193830

[B10] Hollis J. L. Williams A. J. Sutherland R. Campbell E. Nathan N. Wolfenden L.. (2017). A systematic review and meta-analysis of moderate-to-vigorous physical activity levels in elementary school physical education lessons. Int. J. Behav. Nutr. Phys. Act. 14, 52. 10.1186/s12966-017-0504-0 28438171 PMC5402678

[B11] Howe C. A. Freedson P. S. Feldman H. A. Osganian S. K. (2010). Energy expenditure and enjoyment of common children's games in a simulated free-play environment. J. Pediatr. 157, 936–942. 10.1016/j.jpeds.2010.06.041 20708746

[B12] Jago R. Macdonald-Wallis C. Solomon-Moore E. Thompson J. L. Lawlor D. A. Sebire S. J. (2017). Associations between participation in organised physical activity in the school or community outside school hours and neighbourhood play with child physical activity and sedentary time: a cross-sectional analysis of primary school-aged children from the UK. BMJ Open. 7, e017588. 10.1136/bmjopen-2017-017588 28912195 PMC5640140

[B13] Janz K. F. Dawson J. D. Mahoney L. T. (2000). Tracking physical fitness and physical activity from childhood to adolescence: the Muscatine study. Med. Sci. Sports Exerc. 32, 1250–1257. 10.1097/00005768-200007000-00011 10912890

[B14] Kelly L. A. Reilly J. J. Jackson D. M. Montgomery C. Grant S.. (2007). Tracking physical activity and sedentary behavior in young children. Ped. Exerc. Sci.19, 51–60. 10.1123/pes.19.1.51 17554157

[B15] Khawaja I. Woodfield L. Collins P. Benkwitz A. Nevill A. (2020). Tracking children's physical activity patterns across the school year: A mixed-methods longitudinal case study. Children. 7, 0178. 10.3390/children7100178 33053815 PMC7600523

[B16] Malina R. M. (1996). Tracking of physical activity and physical fitness across the lifespan. Res. Q. Exerc. Sport. 67, 48–57. 10.1080/02701367.1996.10608853 8902908

[B17] Malina R. M. (2001). Tracking of physical activity across the lifespan. Res. Digest. Pres. Counc. Phys. Fit. Sport. 14, 1–10. 10.1037/e603482007-0018902908

[B18] McMurray R. G. Harrell J. S. Bangdiwala S. I. Hu J. (2003). Tracking of physical activity and aerobic power from childhood to adolescence. Med. Sci. Sports Exerc. 55, 1914–1922. 10.1249/01.MSS.0000093612.59984.0E 14600559

[B19] Moghaddaszadeh A. Ahmadi Y. Belcastro A. N. (2016). Children and adolescent physical activity participation and enjoyment during active play. J. Sports Med. Phys. Fitness. 57, 1375–1381. 10.23736/S0022-4707.16.06732-3 28004904

[B20] Moghaddaszadeh A. Belcastro A. N. (2021). Active play promotes physical activity and improves fundamental motor skills for school-aged children. J. Sport Sci. Med. 20, 86–93. 10.52082/jssm.2021.86 33707991 PMC7919351

[B21] Moghaddaszadeh A. Jamnik V. Belcastro A. N. (2018). Characteristics of children's physical activity during active play. J Sports Med Phys Fitness. 58, 369–376. 10.23736/S0022-4707.16.06704-9 27735889

[B22] Munoz S. R. Bangdiwala S. I. (1997). Interpretation of kappa and B statistics measures of agreement. J. Appl. Stat. 24, 105–112. 10.1080/02664769723918

[B23] Pate R. R. Trost S. G. Dowda M. Ott A. E. Ward D. S.. (1999). Tracking of physical activity, physical inactivity, and health-related fitness in rural youth. Ped. Exerc. Sci. 11, 364–376. 10.1123/pes.11.4.36416015137

[B24] Pettee-Gabriel K. K. Morrow J. R. Woolsey A. L. T. (2012). Framework for physical activity as a complex and multidimensional behavior. J. Phys. Act Health. 9, 11–18. 10.1123/jpah.9.s1.s11 22287443

[B25] Poitras V. J. Gray C. E. Borghese M. M. Carson V. Chaput J. P. Janssen I.. (2016). Systematic review of the relationships between objectively measured physical activity and health indicators in school-aged children and youth. Appl. Physiol. Nutr. Metab. 41, 197–239. 10.1139/apnm-2015-0663 27306431

[B26] Pyle A. Danniels E. (2017). A continuum of play-based learning: The role of the teacher in play-based pedagogy and the fear of hijacking play. Early Educ. Dev. 28, 274–289. 10.1080/10409289.2016.1220771

[B27] Roth A. Schmidt S. C. E. Seidel I. Woll A. Bos K. (2018). Tracking of physical fitness of primary school children in Trier: A 4-year longitudinal study. Biomed. Res. Int. 2018, 1–10. 10.1155/2018/7231818 29850555 PMC5937626

[B28] Truelove S. Vanderloo L. M. Tucker P. (2017). Defining and measuring active play among young children: a systematic review. J. Phys. Act. Heal. 14, 155–166. 10.1123/jpah.2016-0195 27775475

[B29] Verswijveren S. J. J. M. Lamb K. E. Bell L. A. Timperio A. Salmon J.. (2018). Associations between activity patterns and cardio-metabolic risk factors in children and adolescents: A systematic review. PLoS ONE. 13, e0201947. 10.1371/journal.pone.0201947 30114269 PMC6095515

[B30] West S. T. Shores K. A. (2008). A comparison of four recreation styles and physical activty outcomes in elementary school children. J. Park Recrreat. Admin. 26, 115–133.

